# Multigenerational Health Perspectives: The Role of Grandparents’ Influence in Grandchildren’s Wellbeing

**DOI:** 10.3389/ijph.2023.1606292

**Published:** 2023-09-08

**Authors:** Emre Sari

**Affiliations:** ^1^ Division for Health and Social Sciences, NORCE Norwegian Research Centre, Tromsø, Norway; ^2^ School of Business and Economics, UiT The Arctic University of Norway, Tromsø, Norway

**Keywords:** grandparental investment, intergenerational health, public health policy, multigenerational transmission, demographic

**The IJPH series “Young Researcher Editorial” is a training project of the Swiss School of Public Health.**

As populations age, grandparents are playing larger roles in their grandchildren’s lives. Their role has expanded during a time of demographic transition characterized by longer life expectancy, later childbearing years, and declining fertility rates, all of which have changed the structure of families. Though grandparents are increasingly shaping their grandchildren’s health and overall wellbeing, their role is understudied. We must rectify this by expanding our focus, adding grandparents to the parent-child relationship and enabling us to capture the complex dynamics of grandparent-parent-grandchild triad.

The profound effect of grandparents on key aspects of their grandchildren’s development, including cognitive and verbal abilities, mental health, and overall wellbeing, has been covered in Coall and Hertwig’s literature reviews [1]. These reviews discuss research on grandparents’ altruism from disciplinary perspective ranging from evolutionary theory and sociology, to economics. Coall and Herwig suggest that young teenage mothers benefit from a balanced support system in which grandparents assist without usurping the mother’s role. They consider studies from more and less industrialized societies and elucidate the influence factors such as living conditions and societal norms play in shaping grandparents’ roles. For example, in larger families, resources for children might be limited by the need to divide them among so many family members. Likewise, the presence of other kin, and a grandchild’s birth order, can influence the degree of closeness in the relationship between grandparent and grandchild [1].

The broad dimensions of grandparents’ investment are discussed by Buchanan and Rotkirch [2], who focus on economic, emotional, and social aspects of the relationship. They explore various influences, including human evolution, that have shaped grandparents’ behavior and examine the effects of lineage, gender, and marital status on grandparenting. Breen [3] illuminates methodological issues in multigenerational studies that strive to determine the direct influence of grandparents on their grandchildren. While this editorial also focuses on the influence grandparents exert on their grandchildren, Wong et al. [4] reminds us that this influence is bidirectional. In this instance, I will encourage researchers to study the often underappreciated influence grandparents exert on their grandchildren’s health outcomes.

The nurturing bond between grandparents and grandchildren is likely to influence determinants of child health, and has the potential to reduce risky behavior, including smoking and drug use among teenagers. A strong bond may improve nutritional outcomes, reduce the likelihood of childhood obesity, and be associated with better mental health in grandchildren [5]. This bond can also adversely affect grandchildren, e.g., when grandparents indulge in unhealthy habits, such as smoking [6]. These effects may vary greatly across contexts; for example, in all 33 OECD member countries, co-residence with grandparents has been linked to adverse cognitive and non-cognitive outcomes, such as lower mathematics scores and changes in locus of control [5, 7]. Research underscores the nuanced roles grandparents play in grandchildren’s health, and suggests some families may benefit from interventions [1, 5] designed to amplify the positive influence of grandparents while mitigating potential negatives.

Researchers should study grandparents’ influence on grandchildren’s health across diverse socioeconomic and cultural contexts, including Low-and Middle-Income Countries (LMICs). These studies should consider the different contexts of grandparenting in developed and developing nations and gendered aspects of caregiving. Research on the challenges of grandparenting in LMICs could expand our understanding of grandparental influence on multigenerational health. To conduct this research, we need high-quality datasets and we must devise better data management strategies for cross-generational studies. Data-driven projects like “The Great Leap” [8] and similar projects use interdisciplinary approaches to address this data gap at various levels. As Breen [3] notes, methodological issues such as cultural variations, diverse family structures, and time complexities require careful navigation for accurate insights in multigenerational studies. Since this research needs to be conducted across a broad range of contexts, researchers who devise interventions should keep in mind that while interventions that rely on technological tools (e.g., internet access) and other resources (e.g., transportation) may improve interactions between grandparents and their grandchildren, these benefits may not be equally accessible.

We must stop underestimating the influence of grandparents on their grandchildren’s health and extend our research beyond its focus on parent-child dynamics. Instead we must adopt multigenerational approaches to amplify the positive effects of health policies and interventions that encourage grandparents’ involvement. Acknowledging the vital role of grandparents in promoting better health outcomes is a crucial step in moving towards a multigenerational perspective that will result in more robust health policies and improve health outcomes for children.
